# Vitamin B12 Deficiency (Un-)Detected Using Newborn Screening in Norway

**DOI:** 10.3390/ijns9010003

**Published:** 2023-01-05

**Authors:** Trine Tangeraas, Ulf W. Ljungblad, Elma Lutvica, Erle Kristensen, Alex D. Rowe, Anne-Lise Bjørke-Monsen, Terje Rootwelt-Revheim, Ingjerd Sæves, Rolf D. Pettersen

**Affiliations:** 1 Norwegian National Unit for Newborn Screening, Division of Pediatric and Adolescent Medicine, Oslo University Hospital, 0424 Oslo, Norway; 2 European Reference Network for Hereditary Metabolic Disorders (MetabERN), 0424 Oslo, Norway; 3 Institute of Clinical Medicine, University of Oslo, Mailbox 1171 Blindern, 0318 Oslo, Norway; 4 Department of Pediatrics, Vestfold Hospital Trust, Mailbox 1068, 3103 Tønsberg, Norway; 5 Medical Faculty, University of Oslo, Mailbox 1171 Blindern, 0318 Oslo, Norway; 6 Department of Medical Biochemistry, Oslo University Hospital, 0424 Oslo, Norway; 7 Department of Medical Biochemistry and Pharmacology, Haukeland University Hospital, 5021 Bergen, Norway; 8 Department of Clinical Science, University of Bergen, 5007 Bergen, Norway

**Keywords:** B12 deficiency, newborn screening, dried blood spot, sensitivity, specificity, methylmalonic acidemia, propionic acidemia

## Abstract

Untreated vitamin B12 (B12) deficiency may cause delayed development in infants. Several newborn screening (NBS) programs have reported an increased detection rate of B12 deficiency when second-tier dried blood spot (DBS) analyses of total homocysteine (tHcy) and methylmalonic acid (MMA) are included. This is a retrospective study of newborns reported from NBS during 2012–2021 with confirmed B12 deficiency. DBSs were retrieved from the NBS biobank for second-tier MMA and tHcy analysis. Thirty-one newborns were diagnosed with B12 deficiency out of 552970 screened. Twenty-five were ascertained from sixty-one false positive (FP) cases of methylmalonic acidemia and propionic acidemia (PA), and six infants screened positive for other NBS metabolic diseases with propionylcarnitine (C3) in the normal range. In the original DBS, 7/23 (30%) and 12/23 (52%) of B12-deficient newborns with FP methylmalonic acidemia/PA had MMA and tHcy > 99th percentile. B12 deficiency was a common differential diagnosis of screening positive for methylmalonic and PA. C3 failed to identify a subset of newborns with B12 deficiency. Second-tier MMA and tHcy analyses in the DBS showed suboptimal sensitivity for identifying infants with B12 deficiency. The shortcomings of NBS should be acknowledged when considering B12 deficiency as a primary target of NBS panels.

## 1. Introduction

**Figure 1:**
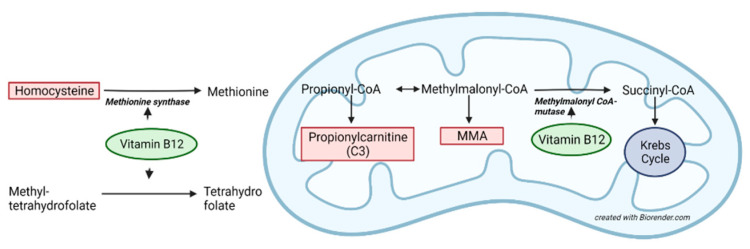
The two vitamin B12-dependent pathways (simplified) in the cytosolic and mitochondrial compartments of the cell. MMA; methylmalonic acid.

The vitamin B12 (B12) level in newborns correlates with maternal B12 levels, and breastfed newborns are at risk of developing B12 deficiency if the mother is vitamin B12 deficient [1,2,3]. Newborns with B12 deficiency are commonly asymptomatic [4,5]. Initial symptoms such as feeding difficulties, hypotonia, tremor, apneas and seizures appear between 1 and 6 months of age [6,7]. The condition can be clinically challenging to distinguish from other common disorders in infancy, and the diagnosis is often delayed [8]. In most cases, B12 deficiency is reversible using supplementation of B12 but, if left untreated, it may lead to permanent developmental delay [9,10]. The cytosolic remethylation of homocysteine to methionine (Met) and the mitochondrial methylmalonyl-coenzyme A mutase, which converts methylmalonyl-CoA to succinyl-CoA, both require B12 as a cofactor (Figure 1). Diminished activity of the former pathway decreases methionine while homocysteine accumulates, and myelinization and numerous methylation reactions are affected. Reduced activity of methylmalonyl-CoA mutase leads to increased methylmalonic acid (MMA) and up-stream propionyl-CoA, which is further converted to propionylcarnitine (C3), the main primary biomarker for methylmalonic and propionic acidemia (PA) in newborn screening (NBS). Maternal B12 deficiency in the first trimester has been reported to be as high as 60% in low-income countries due to poor socioeconomic status and a diet low in animal products, whereas in high-income countries such as Canada, 5% of women in early pregnancy are B12 deficient [11]. A previous study showed that approximately half the pregnant women had insufficient B12 reserves at week 18 of pregnancy to sustain a proper B12 level in their breastfed infant during the first 6 months of life [12]. Even so, no guidelines on actionable limits for B12 deficiency nor a uniform B12 screening policy during pregnancy has been implemented [13]. After delivery, newborns may reveal maternal B12 deficiency using NBS. For more than a decade, NBS programs have reported the identification of B12 deficiency secondary to screening for methylmalonic acidemia and PA with incidences spanning from 0.88 to 33.1/100,000 [14,15,16]. As C3 and C3/acetylcarnitine (C2) are unspecific markers both for the metabolic screening conditions and B12 deficiency, the introduction of the second-tier MMA method in DBS has increased specificity for methylmalonic acidemia and B12 deficiency in NBS [17,18,19]. Recently, several NBS programs have also introduced remethylation disorders as part of their panels. The adjustments of screening algorithms with first-tier markers from both B12-dependent pathways followed with combined second-tier tHcy and MMA have created an opportunity to detect even more newborns (1:1989–1:3300) prone to develop symptomatic B12 deficiency [4,20,21] than by using MMA alone [22]. As B12 deficiency complies with the Wilson and Jungner criteria in that the newborns are asymptomatic upon detection and treatment is effective, inexpensive and easy [23], B12 deficiency has been proposed as a primary target of NBS as the first acquired, diet-dependent condition [24,25].

The purpose of this study was to evaluate the Norwegian NBS experience of B12 deficiency verified after referral of newborns with elevated biomarkers for methylmalonic acidemia and PA and other metabolic screening conditions from the NBS program. We also set out to retrospectively investigate the sensitivity of the second-tier MMA and tHcy in the original DBS as functional markers of B12 deficiency.

## 2. Materials and Methods

### 2.1. Design and Study Population

**Figure 2:**
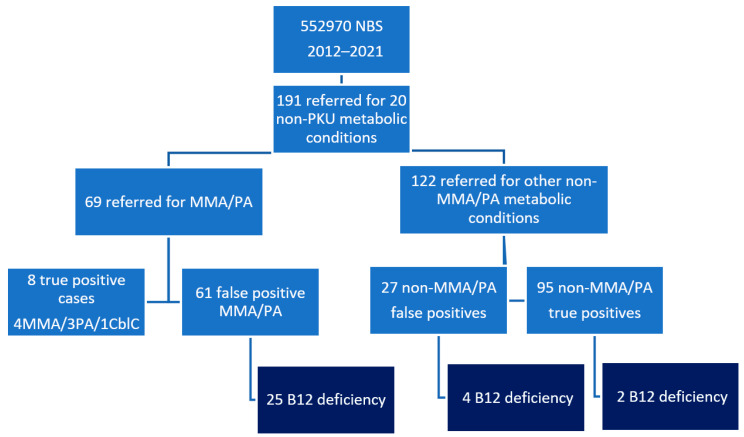
Flowchart showing B12 deficiency detected using NBS from non-PKU metabolic diseases 2012–2021. PKU, phenylketonuria; MMA, methylmalonic acidemia; PA, propionic acidemia; Cbl C, cobalamin C deficiency.

This was a retrospective study of newborns reported from the newborn screening program 2012–2021 in Norway with either false positive (FP) results for methylmalonic acidemia and PA, or newborns reported for other metabolic screening conditions, where confirmatory biochemical and metabolic tests revealed B12 deficiency (Figure 2). Newborns referred for possible methylmalonic acidemia or PA were admitted the same or the following day to the local pediatric department. Metabolic tests of blood and urine were performed at Oslo University Hospital to confirm or exclude the screening disease. B12, MMA and tHcy of the newborn and the mother were part of the standard metabolic work-up procedure and either analyzed at the local hospital or at Oslo University Hospital. According to the protocol, 1 mg of intramuscular hydroxycobalamin was to be administered pending analytical results [26]. If the metabolic confirmatory tests were negative and B12 deficiency was confirmed, further treatment and follow-up were organized at the local pediatric department. The original NBS and metabolic confirmatory results were reviewed using the NBS electronic data laboratory system. The remaining B12 status (B12 or holotranscobalamin, MMA, tHcy and folate) of the child and mother was retrieved from the written feedback report from the local hospital, which also included a clinical evaluation of the infant. Missing or incomplete data were collected using telephone calls from the local pediatrician. For the two cases with delayed development referred to follow-up at our hospital, clinical data were retrieved from the medical record. This study was approved by the Institutional Review Board (IRB) of Oslo University Hospital (20/18182) 26.08.2020. Permission to identify NBS study cases using the national newborn screening registry and to access NBS and confirmatory metabolic results from the electronic data laboratory system were included in the approval from the IRB. This study was registered in accordance with the data protection regulations at Oslo University Hospital.

### 2.2. Newborn Screening DBS Analyses

Blood samples were collected on filter cards 48–72 h after birth and sent using prioritized mail to the Norwegian National NBS laboratory at Oslo University Hospital as previously described [27]. C3 > 4.75 µmol/L and C3/C2 > 0.24 were the primary markers and cut-offs for methylmalonic acidemia and PA, respectively [27]. Acylcarnitines were analyzed from DBS (3.2 mm punch) with the NeoBase^TM^ 2 Non-Derivatized MSMS Kit (PerkinElmer, Turku, Finland) on an Acquity UPLC coupled to a Xevo TQS-micro mass spectrometer (Waters, Milford, MA, USA). After the regular NBS analyses were performed, the DBS screening filter cards were stored for 1–3 months at 2–4 °C before being moved to long-term storage in a biobank at −20 °C. DBS methylmalonic acid (DBS MMA), DBS-total homocysteine (DBS tHcy) and DBS 2-methylcitrate (DBS MCA) were also analyzed in the original DBS. A combined analysis for second-tier tHcy, MMA and MCA was developed for DBS using an LC-MS/MS method adapted from Fu et al. [28] and described in [29]. The method was implemented as a routine second-tier test for C3 and C3/C2 above cut-off during 2020. Normal DBS tHcy, DBS MMA and DBS MCA percentiles were determined from assessments of 450 randomly selected DBS that were sampled and analyzed in batches in 2021. DBS from a selected group of 89 healthy newborns born between 2012 and 2018, who were sampled and matched for another study of B12 deficiency in infants, were also included as controls [29].

### 2.3. Biochemical Analyses

B12, folate and tHcy were analyzed in plasma using electro chemiluminescence immunoassay (ECLIA), Roche Cobas 8000 (e801 og c702, Roche Diagnostics GmbH, Mannheim). Holotranscobalamin assessments were performed using chemiluminescence Immunoassay (Alinity, Abbott Molecular Diagnostics, Des Plaines, IL, USA). Serum MMA was quantified using liquid chromatography-mass spectrometry MMA (AB Sciex QTRAP 4500). Qualitative urinary organic acid analysis was performed using gas chromatography-mass spectrometry (Agilent Intuvo 9000 GC System, Agilent 5977B series MSD).

### 2.4. Definitions

B12 deficiency in newborns was defined as established B12 deficiency (serum or plasma tHcy > 10 µmol/L and B12 < 200 pmol/L) or as functional B12 deficiency (serum or plasma tHcy > 10 µmol/L and B12 > 200 pmol/L), based on the discussions of Hannibal et al. [30]. Maternal B12 deficiency was defined as the combination of B12 < 300 pmol/L and MMA > 0.3 µmol/L [3,31]. Holotranscobalamin < 32 pmol/L combined with an increased functional marker (tHcy > 10 µmol/L in the newborn and MMA > 0.3 µmol/L in the mother) was also categorized as B12 deficiency. Folate adequacy was defined as >20 nmol/L in newborns and >7 nmol/L in adults.

### 2.5. Statistics

Data are presented as median, interquartile range [IQR] (for n > 10) and total range. Categorical variables are presented as proportions and percentages. Comparisons between groups were performed using the Mann–Whitney U test for continuous variables and Chi-Square for categorical variables. Spearman’s rank correlation was applied for continuous non-normally distributed parameters. Second-tier percentiles (DBS MMA, DBS MCA and DBS tHcy) from 450 DBS controls were calculated using Excel 2016 (PERCENTILE.EXE function). The statistical software IBM SPSS Statistic version 26 (IBM Inc., New York, NY, USA) was used for analysis. Two-sided tests were used, and *p* < 0.05 was considered statistically significant.

## 3. Results

### 3.1. Cohort of 61 False Positive Methylmalonic Acidemia and PA

Of 552,970 screened newborns, 61 infants (1:8014) were reported with false positive results for methylmalonic acidemia and PA at a median 6 [5,6,7,8] (3–68) (median [IQR] (range)) days after birth. Fifty-seven were referred after abnormal first-tier tests only, and four infants born in 2020–2021 were referred following the first- and second-tier DBS tHcy and DBS MMA. In total, 25 (41%) showed B12 deficiency (4.5/100000), established in 15/61 (25%) and functional in 10/61 (16%). Descriptive data of the infants and mothers categorized as with or without B12 deficiency are shown in Table 1. All infants but one were asymptomatic. The vast majority showed elevated MMA based on quantitative urine organic acid analysis (Table 1). In a single case, B12 deficiency was only confirmed following a new DBS sample that was requested due to the poor sample quality of the initial DBS. The second DBS test was first sampled on day 68 and received on day 72 after birth at a time when the infant showed poor weight gain (C3 7.7, C3/C2 0.32, DBS tHcy 13 µmol/L and DBS MMA 3.1 µmol/L). Samples for B12 status and metabolic work-up were obtained at a median of 7 days [5,6,7,8] (3–68) after birth, concomitantly with most of the mothers in 51/55 (93%). B12 and MMA data were available for 55/61 (90%, holotranscobalamin in n = 2) and 49/61 (80%) of the mothers, respectively (Table 1). In total, 36% of mothers and infants had their B12 status analyzed at Oslo University Hospital, while the remaining tests were performed at their respective local hospitals. Analyses of B12, tHcy and MMA were complete in 57/61 (93%) of the newborns. Four infants with missing values were categorized without B12 deficiency since one of tHcy, MMA or B12 was within the normal values and since none had MMA detected in the urine. None of the newborns were folate deficient (analyzed in 42/61, 69%). Metabolic confirmatory tests (acylcarnitines or urine organic acids) were missing or incomplete for nine newborns including four preterm babies. The mothers of newborns categorized as B12-deficient had significantly higher tHcy than mothers of newborns without B12 deficiency (Table 1). Of the 25 B12-deficient infants and the 36 infants without B12 deficiency, 12/19 (63%) and 9/30 (30%) of the mothers were categorized as B12-deficient, respectively (Chi square, *p* = 0.02). For the mothers to infants with B12 deficiency, 3/21 had B12 < 100 pmol/L, 12/21 (57%) B12 100–200 pmol/L and 6/21 (29%) had B12 200–300 pmol/L. The correlation between maternal B12 tests and her infant tHcy was for total B12 rho = 0.36 (*p* = 0.01), for MMA rho = 0.47 (*p* = 0.001) and for tHcy rho = 0.57 (*p* < 0.001). There were no correlations between the serum folate of mothers and infants.

#### DBS Second-Tier Analysis

The original first-tier DBS biomarkers for the 61 false positive newborns and 89 controls are shown in Table 2. There were no significant differences in C3 or C3/C2 between infants categorized with or without B12 deficiency. Two infants had isolated Met < 5th percentile (11.7 µmol/L), two infants had Met/Phe < 5th percentile (<0.23) and a single infant had both Met and Met/Phe < 5th percentile. The DBS (retrievable in 59/61, 97%) had been stored for a median of 6.3 years [3.2-8-2] (0.02–8.9) after birth before being analyzed for DBS MMA, DBS MCA and DBS tHcy. There were no significant differences in storage time between the groups (Table 2). DBS MMA and DBS MCA were significantly higher in the newborns with B12 deficiency, compared to both those without B12 deficiency and to the 89 DBS controls (Mann–Whitney U test, *p* < 0.001). DBS tHcy was significantly higher in infants with B12 deficiency as compared to controls (Mann–Whitney U test, *p* < 0.001) and to infants categorized with no B12 deficiency (Mann–Whitney U test, *p* = 0.035) (Table 2). Three infants (two with B12 deficiency) had DBS MCA > 99th percentile (>0.97 µmol/L).

Overall, 7 of 23 (30%) and 12/23 (52%) of the false positive methylmalonic acidemia and PA classified as B12 deficient at one week of age exhibited DBS MMA > 1.14 µmol/L (99th percentile) and DBS tHcy > 10.54 µmol/L (99th percentile), respectively. Seven (30%) had combined DBS tHcy and DBS MMA > 99th percentile. In the newborns who were not defined as B12-deficient at one week of age, 2/34 and 8/34 (23%) had DBS MMA and DBS tHcy > 99th percentile. In all 61 infants, DBS tHcy and MMA correlated significantly with plasma tHcy and MMA at one week of age (rho 0.47, *p* < 0.001 and rho 0.68, *p* < 0.001), respectively.

### 3.2. Newborns Reported for Other Metabolic Diseases from NBS Diagnosed with B12 Deficiency

During 2012–2021, a total of 191 newborns with non-PKU metabolic conditions were referred from the Norwegian NBS (Figure 2). In six (3%) newborns (glutaric aciduria type 2, carnitine transporter deficiency, cystathionine β-synthase deficiency and biotinidase deficiency), the standard metabolic work-up at median 10 days of age (3–20) revealed qualitatively increased MMA in urine organic acids, which prompted analysis of B12 status of the infants. B12 was measured at a median (total range) of 125 pmol/L (101–181), MMA was measured at a median of 3.5 µmol/L (2.1–6) and tHcy was measured at a median of 15 µmol/L (11.2–35). B12 status was not available in any of the mothers. A review of the DBS results showed a C3 median of 0.73 (0.59–2.49) and a C3/C2 median of 0.11 (0.06–0.15). None of the six infants had Met or Met/Phe < 5th percentile in the screening sample. DBS MMA and DBS tHcy were measured at a median of 0.3 (0.2–27) and 14 (8.9–47.6), respectively. One infant had MMA > 99th percentile and three infants had tHcy > 99th percentile in analyses of the original DBS.

### 3.3. Two Clinically Ascertained Cases with Severe, Symptomatic B12 Deficiency

Two infants aged eight and nine months were referred to Oslo University Hospital during the study period for metabolic work-up due to delayed development starting at 4 months of age, which progressed to developmental regression and apathy. Both were exclusively breastfed refusing complementary feedings, and both were diagnosed with severe B12 deficiency. Neither had been reported using NBS. Table 3 shows NBS results from specimens sampled at 48–52 h and B12 status in both mother and infant obtained at 8.5 and 9 months of age, respectively. The mothers had normal folate levels of 26 µmol/L and 44 µmol/L. The infants were born prior to the implementation of current second-tier algorithms. The C3/C2 marker would have triggered second-tier tests for DBS tHcy and DBS MMA in one of the two infants, whereas DBS Met and Met/Phe were in the normal range for both infants. Both mothers were asymptomatic and diagnosed with pernicious anemia. During longer-term follow-up, one was diagnosed with attention deficit disorder at 9 years of age and the other was tested with Bayley Scales of Infant and Toddler Development at the age of 2 years and 4 months with a cognitive domain score corresponding to 12–13 months developmental age.

## 4. Discussion

During the 10-year period, 31 newborns (1:17837) were ascertained with B12 deficiency using the expanded NBS in Norway, 25 as a spin-off effect of screening for propiogenic acidemias and another 6 as incidental findings in confirmatory testing for other metabolic screening diseases with DBS C3 and C3/C2 remaining well below the cut-off limits. Despite the lack of DBS MMA and DBS tHcy in the majority of the NBS cases at the time of referral, our results are in accordance with the findings of a NBS study from Wisconsin, yielding the same proportion (~40%) of false positive MMA and PA due to B12 deficiency [19]. NBS in Norway is currently not designed with the intention of screening for infantile B12 deficiency. Thus, the detection rate of identified B12 deficiency at NBS is low compared to the findings in other Scandinavian studies where 0.31–0.34% of infant populations are clinically diagnosed with symptomatic B12 deficiency [7,8]. Moreover, in a Norwegian NBS study performed in 1999, where NBS specimens were obtained as serum samples, 5% of newborns showed B12 deficiency [32]. A similar study is difficult to reproduce as B12 in pmol/L concentration is challenging to measure using DBS, but the results from 20 years ago are still 100 times higher than those reported in recent NBS studies using algorithms adjusted to detect B12 deficiency [4,20,21].

The B12 deficiency in newborns and mothers identified using NBS varies among studies due to different biomarker cut-offs and the availability of second-tier tests. Different maternal diet habits and gastrointestinal risk factors may also play a role. Comparison of incidence between studies is therefore a challenge. Moreover, the case definition of infant B12 deficiency is not clearly established in NBS publications, although most favors the use of the functional markers tHcy and MMA to increase sensitivity and specificity [2,3,19,30]. It is generally agreed that tHcy is the best functional marker of B12 deficiency in newborns [20,21,24,33], hence our definition was chosen accordingly [30]. If an alternative definition derived from a study of four-day-old infants had been applied instead (tHcy > 11 µmol/L or MMA > 0.6 µmol/L, both corresponding to 97.5th percentile) [34], the same proportion (40%) of newborns would have been categorized as B12-deficient among the false positives reported in our study. According to the definition of Refsum et al. (B12 < 200 pmol/L and tHcy > 10 µmol/L or MMA > 0.40 µmol/L and tHcy >10 µmol/L) [32], approximately the same percentage (24/61, 39%) of our cases were B12-deficient. Bjørke-Monsen and coworkers have recommended tHcy < 6.5 µmol/L as the intervention threshold for vitamin-optimized infants [35]. Randomized studies have shown that it is possible to improve neurodevelopmental scores in infants with tHcy > 6.5 µmol/L by providing B12, indicating that even a suboptimal B12 status during this important period of brain development may impair neurodevelopment [36,37]. Two-thirds of the sixty-one newborns would fit into this definition, in accordance with the finding in a supposedly healthy cohort of infants [35].

If the second-tier threshold of the 99th percentile of DBS tHcy had been chosen in our NBS program, only half of the infants with B12 deficiency would have been referred for confirmatory testing. Lowering the DBS tHcy threshold would increase sensitivity at the cost of more false positives, and almost a quarter of cases without defined B12 deficiency had elevated DBS tHcy > 99th percentile. Despite that 60% of the newborns with increased C3 or C3/C2 were not categorized with B12 deficiency, a number of infants could possibly have developed B12 deficiency later as it is well known that B12 diminishes and tHcy increases after the first week in life [33,34,38]. In accordance with this reasoning, a third of the mothers of the group of infants with no B12 deficiency met the criteria for B12 deficiency.

The level of tHcy was an important premise for our chosen B12 definition. As folate deficiency is very rare in infancy [2], other factors such as nitrous oxide (N_2_O) given as maternal pain relief during delivery may transiently increase tHcy levels days after birth by the irreversible inhibition of methionine synthase [39] and possibly confound the interpretation of tHcy both at two days in life and at one week age. In a recent case-control study, the dose of N_2_O during labor was shown to be the strongest predictor for NBS DBS tHcy [8]. The results suggested the use of N_2_O reduces the specificity of tHcy in NBS DBS and may be one of the factors underlying the requirement of a new DBS sample test in NBS programs to increase the positive predictive value of B12 deficiency [4,21,24].

Most of the mothers of newborns with B12 deficiency had B12 levels within the lower-normal range, and 37% of the mothers did not meet our definition of B12 deficiency a week after birth. Other NBS programs have shared the same experience with many mothers having normal or subnormal B12 level [15,16,20]. We can only speculate whether the use of N_2_O indirectly impacted the assignment of an infant as B12-deficient with a B12-replete mother. Unfortunately, we had no data on whether the mothers had received N_2_O as analgesia during labor or not in our study. In addition, it has been proposed that the B12 level in mothers increases after birth, at the expense of intracellular levels, to mobilize B12 to the breastmilk [12,40]. The B12 level postpartum may therefore have been overestimated and affected the proportion of mothers fulfilling the definition of B12 deficiency.

Our study demonstrated that the primary markers C3 and C3/C2 could not distinguish B12 deficiency from other false positives of methylmalonic acidemia and PA. If primary markers of the remethylation pathway (Met and Met/Phe), according to the proposed algorithm of Gramer et al. [5], were to be incorporated into the Norwegian NBS program, about 80 cases would need to undergo second-tier testing (DBS tHcy/MMA/MCA) per week. However, to maintain high sensitivity and specificity, several newborns would need to take a new DBS sample test to show the persistence of elevated DBS tHcy or MMA before being recalled a third time for confirmatory testing [4,20,21]. Adjusting the strategy to include a new DBS sample test in low to moderately increased levels of DBS MMA and DBS tHcy to optimize sensitivity, as completed in a Catalonian NBS study, led to a true positive B12 diagnosis in approximately 11% of the cases [21]. A new DBS sample test is not routine for any NBS condition in Norway, and the implementation may be less feasible in a geographically vast country, disfavoring families that live far from the nearest maternity ward. If remethylation disorders would be included in the Norwegian NBS program in the future, an alternative option could be to increase the action limit for DBS tHcy and MMA to avoid a new DBS sample test, but at the cost of decreased sensitivity for infants and mothers with B12 deficiency.

The limitations to this study were several. First, it was not designed for the purpose of evaluating B12 deficiency secondary to NBS, and data outside the mandate of NBS was not accessible. Therefore, we do not know whether the infant was breast- or formula-fed, nor were the biochemical or clinical outcomes after B12 substitution systematically retrieved. Second, data regarding the mothers’ risk factors or diagnostic work-up for the causes of B12 deficiency remained unknown for most cases. B12 and the functional tests were performed at the local hospital in 2/3 of the cases, and analytical variation in B12 parameters was not adjusted between laboratories. Our cohort was confined to selected cases of false positives and we did not have access to B12 status in an unselected group of infants and their mothers. Despite the comparable storage time, we could not exclude that the control group of infants was biased as they were originally matched for another study of infants with B12 deficiency.

The six infants with B12 deficiency referred for metabolic conditions other than methylmalonic acidemia and PA had normal primary markers from both B12-dependent pathways, demonstrating suboptimal sensitivity of NBS for detecting early B12 deficiency. Moreover, for the two cases presenting with severe B12 deficiency later in infancy, only one would have been referred if second-tier tests had been available at that time. NBS for B12 deficiency may be limited by not detecting all infants presenting later in infancy [24]. Ljungblad et al. showed that, in fact, the majority of infants presenting with B12 deficiency beyond the neonatal period may not be detected using NBS [41].

## 5. Conclusions

NBS can certainly be of benefit for detecting B12 deficiency in the exclusively breastfed infant and her mother to avoid symptomatic B12 deficiency and prevent deficiency in later pregnancies. NBS programs will continue to detect B12 deficiency either systematically or as an adjunct to the regular screening program, and increasingly so if remethylation diseases are introduced in NBS panels. We conclude that NBS for B12 deficiency is not straightforward, and it still remains elusive which factors promote or interfere with the detection of B12 deficiency using NBS at different time points in infancy. We also need to address whether screening and treatment of maternal B12 deficiency during pregnancy is a better preventive strategy to avoid deficiency in both mother and child or at least be complementary to NBS.

## Figures and Tables

**Table 1:** Confirmatory B12 status in newborns reported with false positive results for methylmalonic acidemia and propionic acidemia (n = 61) and their mothers. Categorized with and without B12 deficiency. Numbers are given with median, interquartile range and range. The number in parenthesis is the number of cases with available analyses.

	Newborns without B12 Deficiency (n = 36)		Newborns with B12 Deficiency (n = 25)	
Plasma/serum	Median [IQR]	Range	Median [IQR]	Range
Age at sampling	6 [5–8.9] days	3–14	7 [6–9] days	2–68
	Vitamin B12 pmol/L		Vitamin B12 pmol/L	
Newborns	222 [200–286] (34 ^1^)	112–679	155 [109–223] (24 ^1^)	60–327
Mothers	213 [162–321] (32 ^1^)	90–563	171 [124–255] (21 ^1^)	90–333
	Folate nmol/L		Folate nmol/L	
Newborns	27 [22–31] (23)	10–57	30 [22–36] (19)	17–48
Mothers	13 [9.4–17] (25)	4–41	11 [9.8–16] (19)	3–29
	tHcy µmol/L		tHcy µmol/L	
Newborns	7 [6.1–8] (35)	3.6–10	13.4 [11.1–20] (25)	10–64
Mothers	9 [7.2–11] * (30)	5–16	11.2 [10.0–15] * (21)	7.7–19.3
	MMA µmol/L		MMA µmol/L	
Newborns	0.3 [0.25–0.5] ** (32)	0.1–1.1	1.6 [0.79–6.1] ** (25)	0.4–27.7
Mothers	0.23 [0.18–0.4] (30)	0.1–0.8	0.49 [0.25–0.6] (19)	0.1–3.2
	U-MMA (%) ^a^	-	U-MMA (%) ^a^	-
Newborns	1/31 ^2^ (0.03) **		20/23 ^3^ (87) **	

IQR, interquartile range; tHcy, total homocysteine; MMA, methylmalonic acid; ^1^ Holotranscobalamin was analyzed in two newborns and their mothers, ^a^ urine methylmalonic acid present in proportion of cases using qualitative organic acids analysis, ^2^ missing data in n = 5, ^3^ missing data in n = 2, * Mann–Whitney U test, *p* < 0.05, ** Mann–Whitney U test, *p* < 0.001.

**Table 2:** Newborn screening data in cases reported with false positive results for methylmalonic acidemia and propionic acidemia (n = 61) categorized with and without B12 deficiency and 89 DBS controls. Numbers are given with median, interquartile range and range. The number in parenthesis is the number of cases with available analyses.

Variable	Newborns without B12 Deficiency (n = 36)		Newborns with B12 Deficiency (n = 25)		Controls (n = 89)	
	Median [IQR]	Range	Median [IQR]	Range	Median [IQR]	Range
Gestational age (weeks)	39 [37–40]	27–42	39 [38–40]	28–41	40 [38–41]	(35–42)
Female/male (%)	22/14 (61/39)		15/10 (60/40)	-	46/48 (49/51)	-
Birthweight (g)	3322 [2923–3697]	935–4465	3120 [2745–3797]	1340–4880	3410 [3085–3780]	(2035–4680)
NBS DBSo	Median [IQR]	Range	Median [IQR]	Range	Median [IQR]	Range
C3 (µmol/L)	9.4 [7.2–11.8]	3.3–18.5	7.7 [6–9.9]	3.8–17.2	1.8 [1.4–2.3]	0.7–6.9
C3/C2	0.27 [0.2–0.3]	0.11–0.42	0.26 [0.21–0.3]	0.17–0.60	0.08 [0.06–0.1]	0.04–0.2
Methionine (µmol/L)	20 [17.3–22.0]	12–30	17.9 [14–28]	9–42	17.5 [14.3–20.1]	9.4–32.9
Met/Phe	0.35 [0.3–0.42]	0.22–0.68	0.31 [0.26–0.40]	0.18–0.5	0.34 [0.29–0.38]	0.18–0.57
Age at NBS sampling (hours)	55 [51–61]	48–85	54 [51–66]	48–1644	58.5 [53–64.2]	48–110
Age at reporting (days)	5 [4–7]	3–13	7 [5.5–8.5]	3–72	-	-
NBS DBSs 2021	Median [IQR]	Range	Median [IQR]	Range	Median [IQR]	Range
DBS storage (y)	6.9 [3.9–8.3]	0.2–9.0	6.3 [1.4–8.3] (23)	0.2–8.9	4.2 [3.3–6.2]	(2.2–8.9)
DBS MMA (µmol/L)	0.0 [0.0–0.15]	0.0–0.2	0.36 [0.12–3.1] (23)	0.0–32	0.0 [0–0.09]	0.0–0.9
DBS tHcy (µmol/L	7.9 [6.9–10.4]	4.9–19	11.0 [7.3–14.5] (23)	5.4–46	7.2 [5.9–8.9]	3.6–18.9
DBS MCA (µmol/L)	0.2 [0.08–0.4]	0.0–2.0	0.3 [0.07–0.5] (23)	0.05–1.0	0.0 [0.0–0.05]	0.0–1.3

IQR, interquartile range; NBS, newborn screening; DBS, dried blood spot; NBS DBS^o^, original NBS results; C3, propionylcarnitine; C2, acetylcarnitine; Met/Phe, ratio methionine/phenylalanine; NBS DBS^s2021^, NBS DBS second-tier analysis performed in 2021; y, years; DBS Hcy and DBS MMA, total homocysteine and methylmalonic acid analysed in DBS, respectively; MCA, methylcitrate.

**Table 3:** B12 status and NBS results from two cases diagnosed with severe B12 deficiency at 8 and 9 months of age and their mothers.

Patient (P)	DBS C3 µmol/L	DBS C3/C2	DBS Met µmol/L	DBS Met/Phe	DBS * tHcy µmol/L	DBS * MMA µmol/L	Plasma B12 pmol/L	Plasma tHcy µmol/L	Plasma MMA µmol/L
P1 (f)	2.40	0.14	14.5	0.38	24.1	2.11	60	167	30
Mother P1	-	-	-	-	-	-	60	30	2.0
P2 (m)	3.05	0.40	14	0.24	22.7	41.1	80	110	231
Mother P2	-	-	-	-	-	-	91	66	4

DBS, dried blood spot; Met, methionine; Met/Phe, methionine/phenylalanine; f, female; m, male; * tHcy DBS and MMA DBS were analyzed in 2021 in the original DBS.

## Data Availability

Not applicable.
